# Observations of cold-induced vasodilation in persons with spinal cord injuries

**DOI:** 10.1038/s41393-024-00960-3

**Published:** 2024-02-22

**Authors:** Yasuhisa Fujita, Yoshi-ichiro Kamijo, Tokio Kinoshita, Takamasa Hashizaki, Kouta Murai, Tatsuya Yoshikawa, Yasunori Umemoto, Chikako Kaminaka, Manabu Shibasaki, Fumihiro Tajima, Yukihide Nishimura

**Affiliations:** 1 Department of Rehabilitation Medicine, Wakayama Medical University, Wakayama, Japan; 2 Department of Rehabilitation Medicine, Dokkyo Medical University Saitama Medical Center, Koshigaya, Japan; 3 Department of Dermatology, Wakayama Medical University, Wakayama, Japan; 4 Department of Technology, Nara Women’s University, Nara, Japan; 5 Department of Rehabilitation Medicine, Iwate Medical University, Yahaba-cho, Japan

**Keywords:** Vasodilation, Blood flow

## Abstract

**Study design:**

Acute experimental study.

**Objectives:**

Cold-induced vasodilation is a local mechanism of protection against frostbite in non-injured persons. We assessed whether an increase in skin blood flow (SkBF) during local cooling (LC) was observed in individuals with spinal cord injuries (SCIs) and if the response patterns differed between region levels or sites.

**Setting:**

Laboratory of Wakayama Medical University and the affiliated clinics, Japan.

**Methods:**

A local cooler device (diameter 4 cm) was placed on the chest (sensate) and right thigh (non-sensate) in persons with cervical (SCI_C_; *n* = 9) and thoracolumbar SCIs (SCI_TL_; *n* = 9). After the surface temperature under the device was controlled at 33 °C for 10 min (baseline), LC (−0.045 °C/s) was applied and the skin temperature was maintained at 15 and 8 °C for 15 min of each stage. SkBF (laser Doppler flowmetry) was monitored using a 1-mm needle-type probe inserted into its center.

**Results:**

The percent change in SkBF (%ΔSkBF) on the chest remained unchanged until the end of 15 °C stage; thereafter, it increased to a level at least 70% greater than the baseline during the 8 °C stage in both groups. The %ΔSkBF on the thigh in both SCI_C_ and SCI_TL_ notably increased from 8 and 6 min respectively, during the 8°C stage, compared to 1 min before the stage; however, it did not exceed the baseline level.

**Conclusions:**

An increase in SkBF during LC was observed both in the sensate and non-sensate areas in SCIs, although the magnitude was larger in the sensate area.

## Introduction

Cold-induced vasodilation (CIVD) was first reported by Lewis [1]. CIVD is the physiological phenomenon in which skin temperature on the fingers and toes decreases initially when immersed in cold water <10 °C while the skin temperature increases for 5–10 min after the onset of cooling [2, 3]. This phenomenon prevents frostbite at the acral site [4, 5].

CIVD has been reportedly observed in the fingers, palms, toes, soles, nose, ears, and other areas where arteriovenous anastomoses are abundant [4–7], and its vasomotor control is driven only by the vasoconstrictor components of the sympathetic nervous system of the skin [8]. CIVD has been observed occasionally in the trunk and extremities that are in contact areas subjected to pressure, such as the gluteus maximus, patella, and elbow [9]. Bergersen et al. [10] and Geurts et al. [11] indicated that CIVD was a local reaction not mediated by the central nervous system. Daanen et al. [12] indicated that CIVD was not likely to be induced by the axon reflection of the sensory nerves and local areas but by the vascular endothelial function of the skin [4, 5]. The release of nitric oxide (NO) might be related to this phenomenon. However, it remained unclear whether CIVD would appear in individuals with spinal cord injuries (SCIs).

The sympathetic nervous system is completely segregated from the central nervous system in persons with complete cervical SCIs (SCI_C_), although the disruption to sympathetic control is dependent on the level of the lesion in individuals with thoracolumbar SCIs (SCI_TL_). However, sensory function depends on the level of the lesion in patients with complete SCIs. The sensory nerve is intact on the subclavian area of the chest in persons with SCI_C_ when the level of lesion is lower than C5. As a withdrawal of the sympathetic nervous system directly contributes to modify the CIVD response in the finger [13], if CIVD occurs on the chest and thigh in both SCIs, the response patterns would be different between SCI_C_ and SCI_TL_. If intact sensory nerves influence the skin blood flow (SkBF) responses, these patterns would also differ between the chest and thigh.

We assessed whether response patterns of an increased SkBF would be different on the chest of patients with SCI_C_ and SCI_TL_ and between the chest and thigh in both groups. Local cooling (LC) was performed at 15 °C and 8 °C using a local cooler with a Peltier device. The reasons why the temperature during LC was set at approximately 10 °C were that an increase in SkBF was observed in the acral skin at approximately 10 °C in previous studies [2, 3, 7, 10–13] and our preliminary experiment confirmed that an increase in SkBF was observed even in the non-acral areas of non-injured persons at approximately 10 °C.

## Methods

This acute experimental study was performed in the laboratory of Wakayama Medical University and in clinics that were convenient for participants to visit.

### Participants

Participants were aged ≥20 years and were able to communicate. The inclusion criteria were: (1) persons with SCI_C_ with lesions between levels C5–8 and persons with SCI_TL_ with lesions between levels Th1–L5; (2) the time since injury was > 6 months; and (3) a SCIs classified as A on the American Spinal Injury Association Impairment Scale (AIS); Grade “A” indicates clinically complete injury, suggesting that both motor and sensory functions are compromised at the lesions level [14, 15]. Experienced physiatrists examined the AIS score in each participant. The exclusion criteria included: (1) inflammatory skin diseases including decubitus ulcers, infectious diseases, diabetes or hypertension, dyslipidemia, arteriosclerosis obliterans, collagen disease, liver disease, skin atrophy; (2) those aged ≥65 years, and (3) those receiving steroid therapy. This study was conducted in accordance with the Declaration of Helsinki. Informed consent was obtained voluntarily from all patients before participating in the study. The right to withdraw consent at any time without stating the reason was guaranteed without any individual disadvantage for subsequent medical care. The experimental protocol was approved by the Institutional Review Board for Human Research at the University of Wakayama Medical, Japan (# 2837). The experiment was conducted under the supervision of a physiatrist, with due consideration for safety. The characteristics of the study participants are listed in Table 1.

**Table 1:** Characteristics of participants (SCI_C_ and SCI_TL_).

	Participants	Sex	Age (y)	Height (cm)	Weight (kg)	Lesion level	Time since injury (y)
SCI_C_	1	Male	40	173	52.0	C5	19.0
	2	Male	46	178	55.0	C6	24.0
	3	Male	28	168	58.0	C5	12.0
	4	Male	47	160	35.0	C6	30.0
	5	Male	28	181	61.2	C5	6.8
	6	Male	53	181	80.8	C5	35.0
	7	Male	47	168	57.9	C7	15.5
	8	Male	38	175	41.6	C5	10.3
	9	Male	28	165	51.7	C5	1.1
	Mean ± SD		39 ± 9	172.1 ± 6.9	54.8 ± 12.1		17.1 ± 10.4
SCI_TL_	1	Male	56	172	69.0	L1	8.0
	2	Male	44	170	67.0	Th9	4.0
	3	Female	44	167	57.0	L5	1.0
	4	Male	37	181	64.3	Th7	1.5
	5	Male	34	165	59.9	Th9	15.2
	6	Male	48	170	57.8	Th7	30.8
	7	Male	51	163	68.6	Th10	10.4
	8	Male	28	168	71.9	Th3	1.0
	9	Male	38	163	40.6	Th12	1.0
	Mean ± SD		42 ± 8	168.8 ± 5.2	61.8 ± 9.0		8.1 ± 9.3
	*p* value		0.53	0.29	0.21		0.09

The characteristics of participants with cervical cord injury (SCI_C_) and thoracolumbar spinal cord injury (SCI_TL_) are presented. Lesion level: cervical cord (C), thoracic cord (Th), and lumbar cord (L). Values are presented as the mean ± standard deviation (SD).

### Protocol

**Fig. 1:**
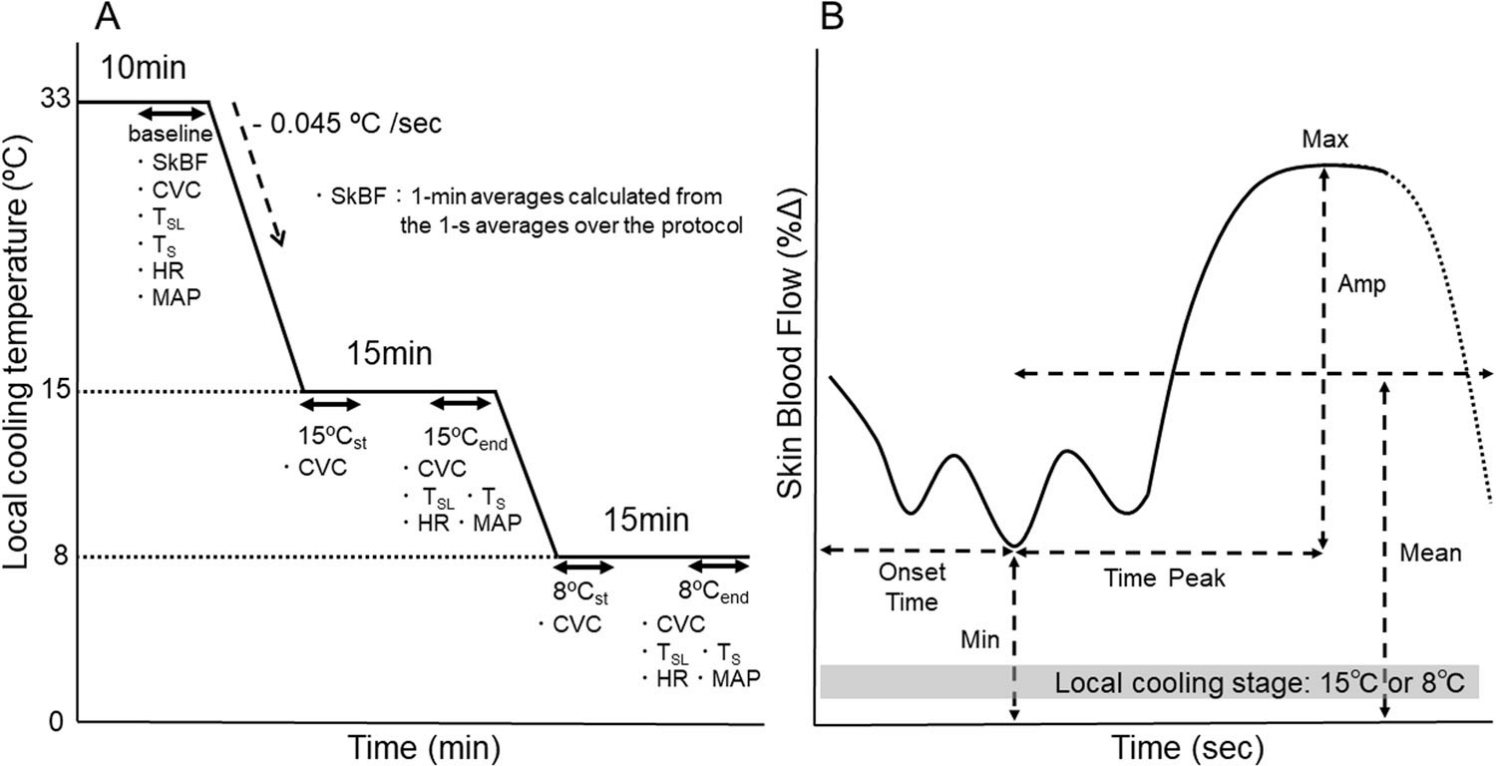
Explanations for a protocol of local cooling and analyses of vasodilation. **A** Protocol of local cooling (LC). The surface temperature of the Peltier device was maintained at 33 °C (baseline) for 10 min, then reduced to 15 °C for 15 min, and then to 8 °C for 15 min. The temperature was decreased from 33 °C to 15 °C and 15 °C to 8 °C at a rate of 0.045 °C/s. Analysis time intervals of measurement items are shown. SkBF (Skin blood flow), CVC (Cutaneous vascular conductance), HR (Heart rate), MAP (Mean arterial pressure), T_SL_ (Sublingual temperature), T_S_ (Mean skin temperature). The baseline value was averaged for the last 5 min of 33 °C. LC of each stage is the first 5 min (15 or 8 °C_ST_) and last 5 min (15 or 8 °C_end_). **B**: Analysis of vasodilator response at the 15 and 8 °C stage. Min (Minimum), Time_onset_ (Time until the Min), Max (Maximum), Time_peak_ (Time to peak), Amp (Amplitude, calculated as Max–Min), Mean (Averaged vasodilation).

The participants were laid on a bed in a room temperature of 25–27 °C and relative humidity of 30–40% for at least 30 min. LC was performed on the right sides of the thigh (L2–5 region) and chest (C4 region) separately in a random order, with intermittent 30 min breaks, except for the first three participants of each group (see Table 1) for whom LC was performed simultaneously on both areas using two Peltier systems. Circular plates of Peltier thermoelectric element, each with 4 cm in diameter, were placed on the skin surfaces, avoiding areas with large veins. The LC area was checked beforehand to determine if participants felt cold. All participants in the SCI_C_ and SCI_TL_ groups felt cold with LC on their chest but not on their thigh. The surface temperature of the Peltier was controlled using a thermoception analyzer (Intercross-210, Intercross Co, Tokyo). The Peltier thermoelectric element had a 1-mm diameter for inserting a needle probe to measure SkBF. As shown in Fig. 1A, the surface temperature of the Peltier was set at 33 °C. After 10 min of rest, the temperature was decreased to 15 °C at a rate of 0.045 °C/s and maintained for 15 min, then decreased to 8 °C at the same rate and was maintained for 15 min.

### Measurements

SkBF was recorded as the blood flow (mV) using a laser Doppler flowmeter (Omega Flow, OMEGAWAVE, Tokyo). A needle-type probe for measuring SkBF was inserted into the 1-mm hole at the center of the Peltier. The blood flow on the skin surface at the LC site was recorded via a 16-bit analog-to-digital converter (AI-1664 LAX-USB, CONTEC, Osaka, Japan) at a sampling frequency of 1000 Hz, with the mean value calculated every 1 s.

A thermocouple (copper-constantan, OMEGA Engineering, Norwalk, CT, USA) was used to measure the sublingual (T_SL_) and skin temperatures at the three sites: the left side of the chest, forearm, and thigh, which were recorded continuously via another analog-to-digital converter (LT-6 Series, Nikkiso Thermo, Tokyo) with a sampling period of 1 s. Mean skin temperatures were calculated as follows with weighted average values: T_S_ = 0.43 × chest + 0.25 × forearm + 0.32 × thigh [16], taking into account the body surface area ratio of each site. A thermocouple sensor was placed between the Peltier device and skin surface.

Heart rate (HR) was calculated from the R–R interval of the electrocardiogram (BSM2401, NIHON KOHDEN, Tokyo). Continuous blood pressure response was measured from the index finger using a beat-by-beat blood-pressure monitoring device (MUB101, Medisence, Tokyo) in the first three participants of each group (see Table 1). The systolic (SBP) and diastolic BPs (DBP) were measured by the auscultatory method using a sphygmomanometer (STBP780; COLIN; Komaki, Japan) every minute in the remaining six participants of each group.

### Analysis

The 1-s mean value of SkBF [mV] was calculated using a commercially available software (The Kissei Comtec; BIMUTUS II; Matsumoto, Japan). The 1-min averages were calculated from the 1-s averages. The decrease in LC temperature from 33 to 15 °C was calculated as an average for the end value 40 s, while the 15 to 8 °C decrease was also calculated as an average for the final value 36 s because the rate of decrease in temperature was 0.045 °C/s, which means that it took 400 s and 156 s for the temperature to decrease from 33 to 15 °C and 15 to 8 °C, respectively. The mean value for the last 5 min of rest at 33 °C was averaged as the baseline, and the percent (%) change (Δ) in SkBF from the baseline was calculated (%ΔSkBF). A comparison between SCI_C_ and SCI_TL_ was made using data obtained from 41 points (1 point as the baseline, 7 points from 33 °C of the local temperature to 15 °C, 15 points from 15 °C LC, 3 points from 15 to 8 °C, and 15 points from 8 °C LC). Characteristics of vasodilation responses during 15 °C and 8 °C LC were assessed as discussed here. The 1-s mean value of SkBF was smoothed by 11-point moving average. The minimum (Min), onset time (Time_onset_), maximum (Max), amplitude (Amp), mean, and time to peak (Time_peak_) of each response were calculated in accordance with the methods reported by Daanen et al. [5] (Fig. 1B): (1) the Min was determined as the lowest value of the %ΔSkBF during each LC stage before the onset of the increase in the %ΔSkBF, (2) the Time_onset_ was the time since the onset of each LC stage until the onset of the increase in the %ΔSkBF [s], (3) the Max was the highest value of the %ΔSkBF, (4) the Amp was calculated as Max–Min, (5) the mean was the averaged value of the %ΔSkBF, and (6) the Time_peak_ was calculated from the onset of each increase to Max. An increase in SkBF was noted in participants where the %ΔSkBF exceeded over 50% of Min.

The T_SL_ and T_S_ were calculated using the 1-min averages. The SBP and DBP were detected from the continuous blood pressure wave; then, the MAP was calculated as (SBP - DBP)/3 + DBP. The HR and MAP were averaged every minute only during the baseline and 15 and 8 °C stages (31 points; 1 point as the baseline, 15 points from 15 °C LC, and 15 points from 8 °C LC). The mean values of the HR and MAP at rest were the mean values during the last 5 min of rest. Cutaneous vascular conductance (CVC = SkBF/MAP) was calculated every minute and averaged for 5 min at baseline and for the first and last 5 min of each LC stage (Fig. 1A; baseline, 15 °C_st_, 15 °C_end_, 8 °C_st_, 8 °C_end_); moreover, the %ΔCVC from the baseline was calculated. The MAP using a sphygmomanometer in six of each group and that using continuous blood pressure wave in three of each group were used to calculate CVC.

### Statistical analyses

Participant characteristics in Table 1 were analyzed using an unpaired t-test. Analyzed using two-way analysis of variance (ANOVA), the %ΔSkBF, T_SL_, and T_S_ were repeatable factors for time (41 levels; baseline, decreasing from 33 to 15 °C, 15 °C stable, decreasing from 15 to 8 °C, 8 °C stable) and factors between groups (2 levels; SCI_C_ and SCI_TL_). The HR and MAP were analyzed as repeatable factors for time (31 levels; baseline, 15 °C stable, 8 °C stable) and in groups (2 levels) from the baseline. CVC was a repeatable factors for time (5 levels; baseline, 15 °C_st_, 15 °C_end_, 8 °C_st_, 8 °C_end_) and the factors between groups were 2 levels. The characteristics of %ΔSkBF response were also tested by two-way ANOVA for repeated measurements; the repeatable factors were temperature (2 levels; 15 °C and 8 °C LC) and the factor between groups (2 levels). The Bonferroni method was applied as a post-hoc test to assess significance in changes during LC, both from the baseline value and from the level measured 1 min before the onset of each stage. The number of participants in whom increased %ΔSkBF was observed were tested by chi-square at 15 °C and 8 °C LC. Statistical significance was set at *p* < 0.05. A statistical power of the *F* test (ANOVA: repeated measure within factors) was calculated using G*Power 3.1.9.2 (Heinrich-Heine University, Düsseldorf, Germany) assuming an α error of 0.05. Effect sizes were calculated using the variances explained by the effect and within-group variances. All analyses were performed using SPSS ver. 25 (IBM, Armonk, NY, USA). All values were expressed as means ± standard deviations (means ± SDs).

## Results

There were no significant differences in age, height, weight, and time since injury between the groups (Table 1).

### SkBF [mV] and CVC [mV/mmHg] on the chest and thigh

**Fig. 2:**
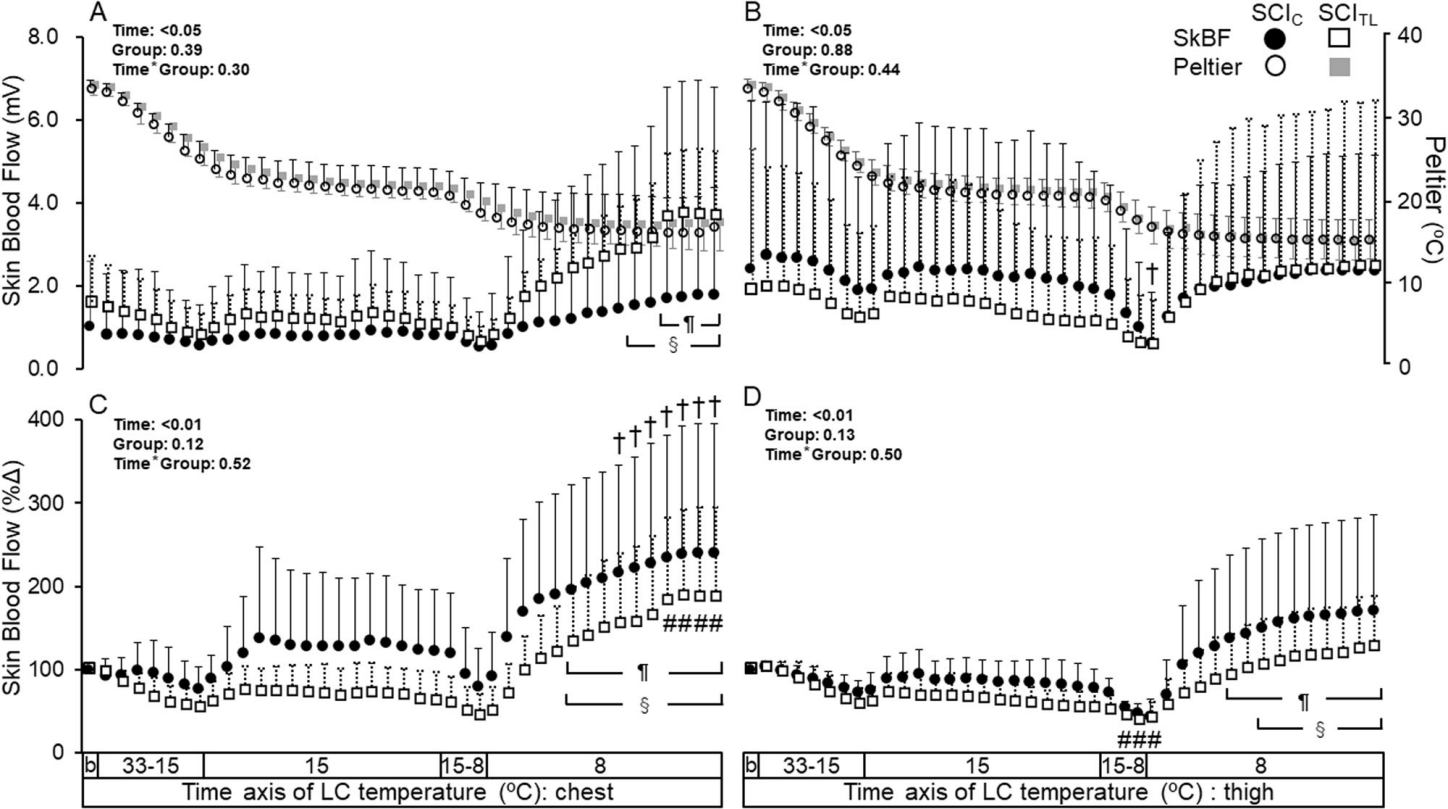
Skin blood flow during local cooling (LC) is shown as the mean ± SD in cervical (SCI_C_; *n* = 9; ●) and thoracolumbar spinal cord injuries (SCI_TL_; *n* = 9; □). The lateral axis shows the time, with baseline, 33 °C to 15 °C, 15 °C stage, 15 °C to 8 °C, and 8 °C stage. **A**: mV value on the chest, **B**: mV value on the thigh, A and B: temperature change between the Peltier device and skin during LC is shown as the mean ± SD in SCI_C_; *n* = 9; ○ SCI_TL_; *n* = 9; ■. **C**: % change from the baseline value (%Δ) on the chest, **D**: %Δ on the thigh. The averaged values for the last 5 min of 33 °C was set as the baseline (0 min). Symbols represent statistical significance: †, vs baseline; §, vs 1 min before the onset of each stage (15 and 8 °C LC); SCI_C_ #, vs baseline; ¶, vs 1 min before the onset of each stage (15 and 8 °C LC); SCI_TL_ at the level of *p* value < 0.05. The results of two-way ANOVA repeated measurements of SkBF are shown in the upper left of the figure. [mV] on the chest and thigh were as follows: effects within participants (Time), F (40, 640) = 6.039 and 3.576, *p* = 0.02 and 0.04; between participants (Group), F (1, 16) = 0.79 and 0.02, *p* = 0.38 and 0.88; interaction effects of Time × Group, F (40, 640) = 1.20 and 0.84, *p* = 0.29 and 0.44, respectively. The results of two-way ANOVA repeated measurements of SkBF [%Δ] on the chest and thigh were as follows: effects within participants (Time), F (40, 640) = 15.29 and 9.08, *p* < 0.001 and 0.004 between participants (Group), F (1, 16) = 2.64 and 2.57, *p* = 0.12 and 0.13; and interaction effects of Time × Group, F (40, 640) = 0.63 and 0.58, *p* = 0.52 and 0.50, respectively.

**Fig. 3:**
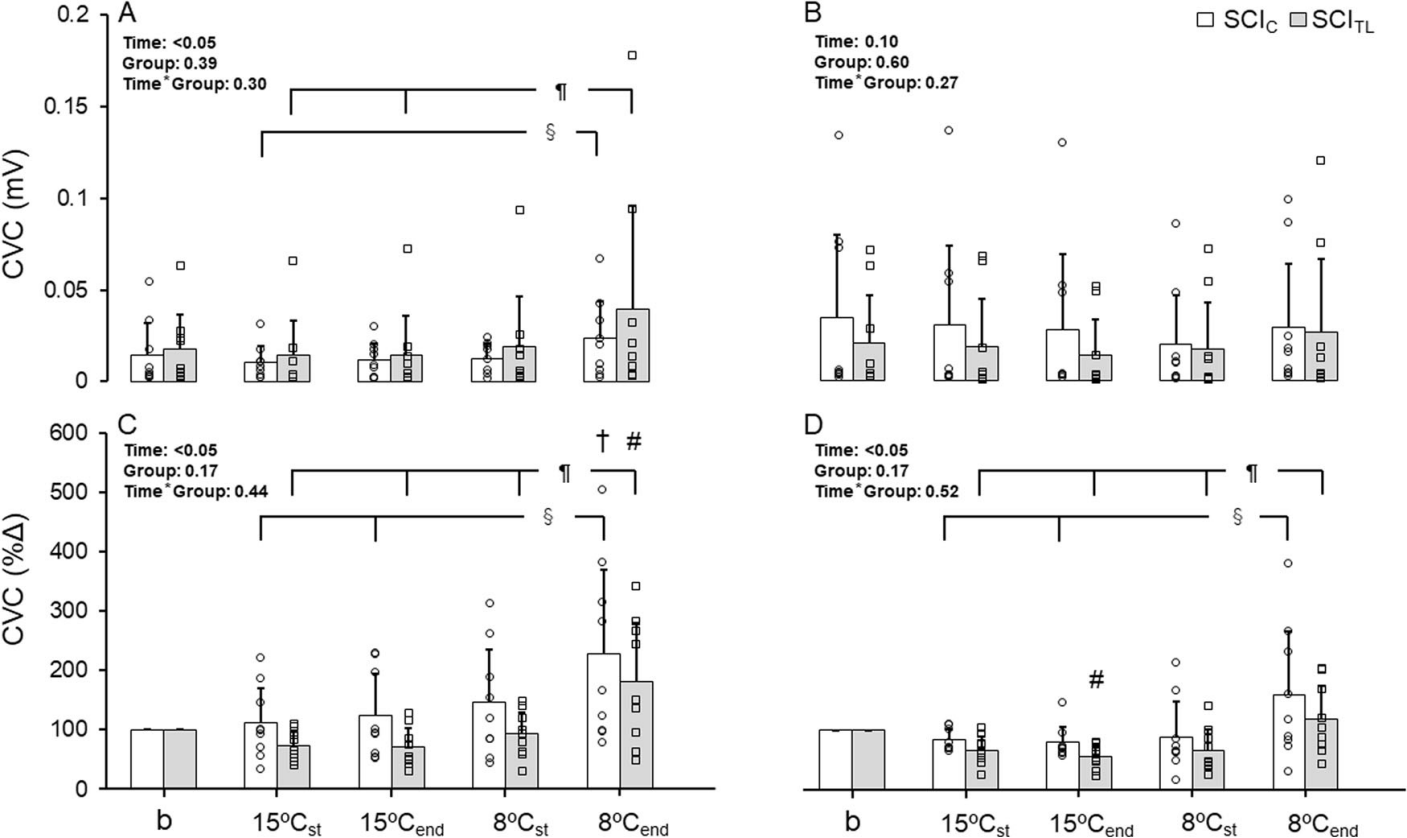
Cutaneous vascular conductance (CVC) during local cooling (LC) is shown as the mean ± SD in cervical (SCI_C_; *n* = 9; □) and thoracolumbar spinal cord injuries (SCI_TL_; *n* = 9; ). **A**: mV/mmHg on the chest, **B**: mV/mmHg on the thigh, **C**: % change from the baseline value (%Δ) on the chest, **D**: %Δ on the thigh. The averaged values for the last 5 min of 33 °C was set as the baseline, LC of each stage is the first 5 min (15 or 8 °C_ST_) and last 5 min (15 or 8 °C_end_). Symbols represent statistical significance: †, vs baseline; §, vs 8 °C_end_ stage (SCI_C_); #, vs baseline; ¶, vs 8 °C_end_ stage (SCI_TL_) at the level of *p* value < 0.05. The results of the two-way ANOVA for repeated measurements of CVC [mV/mmHg] on the chest and thigh were as follows: effects within participants (Time), F (4, 64) = 5.78 and 2.56, *p* = 0.02 and 0.10; between participants (Group), F (1, 16) = 0.35 and 0.29, *p* = 0.56 and 0.60; and interaction effects of Time × Group, F (1, 64) = 0.79 and 1.37, *p* = 0.41 and 0.27, respectively. The results of the two-way ANOVA of %ΔCVC on both sites were as follows: effects within participants (Time), F (4, 64) = 13.55 and 8.52, *p* = 0.001 and 0.005; between participants (Group), F (1, 16) = 2.11 and 2.10, *p* = 0.16 and 0.17; and interaction effects of Time × Group, F (4, 64) = 0.77 and 0.53, *p* = 0.44 and 0.52, respectively.

SkBF [mV] on the chest in both SCI_C_ and SCI_TL_ significantly increased from 1 min before the onset of 8 °C LC during 10 to 15 min and 12 to 15 min at 8 °C LC, respectively (Fig. 2A). SkBF on the thigh in SCI_C_ significantly decreased from the baseline for the first minute of 8 °C LC (Fig. 2B), then returned to the baseline level at 8 °C LC. The statistical powers (1 - β) of the F test of SkBF on the chest in SCI_C_ and SCI_TL_ were 0.98 (effect size 0.26) and 0.99 (0.28), that on the thigh were 0.22 (0.11) and 0.46 (0.15), respectively. CVC levels on the chest and thigh did not significantly deviate from the baseline in both SCI_C_ and SCI_TL_ (Fig. 3A, B). The 8 °C_end_ levels on the chest were significantly higher than the 15 °C_st_ in SCI_C_ and the 15 °C_st_ and 15 °C_end_ in SCI_TL_ (Fig. 3A). The CVC remained constant on the thigh (Fig. 3B).

### %ΔSkBFs and %ΔCVC on the chest and thigh

The %ΔSkBFs on the chest in SCI_C_ significantly increased over the baseline from 9 to 15 min of 8 °C LC, while that in SCI_TL_ increased only in the last 4 min of 8 °C LC compared with the baseline (Fig. 2C). The %ΔSkBF on the thigh in SCI_TL_ significantly decreased from the baseline value of the last 2 min during the decreasing phase from 15 to 8 °C and the first minute at 8 °C LC (Fig. 2D). The %ΔSkBF in both SCI_C_ and SCI_TL_ significantly increased from 8 and 6 min, respectively, to the end of the 8°C LC stage, compared to the level 1 min before the onset of 8 °C LC (Fig. 2D). The statistical powers (1 - β) of the F test of the %ΔSkBF on the chest in SCI_C_ and SCI_TL_ were 0.99 (effect size 0.28) and 0.99 (0.36), that on the thigh were 0.99 (0.28) and 0.99 (0.28), respectively. The %ΔCVC levels on the chest in SCI_C_ and SCI_TL_ significantly increased from the baseline at the 8 °C_end_ (Fig. 3C). The 8 °C_end_ levels on the chest and thigh were significantly higher than the 15 °C_st_ and 15 °C_end_ in both groups (Fig. 3C, D). The %ΔCVC on the thigh in SCI_TL_ significantly decreased from the baseline value at the 15 °C_end_ (Fig. 3D).

### Characteristics of an increased %ΔSkBF response

There were no significant differences in the Time_onset_ between 15 °C and 8 °C LC and between SCI_C_ and SCI_TL_ on both sites (Table 2). The Min in both groups were significantly lower in 8 °C LC than in 15 °C LC on the thigh, while that on the chest remained unchanged in SCI_C_. The Amp in SCI_C_ and SCI_TL_ were significantly higher in 8 °C LC than those in 15 °C LC on both sites (*p* < 0.05 and 0.01, respectively) with no difference between groups and sites. The mean in both groups were higher in 8 °C LC than in 15 °C LC only on the chest (all *p* < 0.05). The Time_peak_ in SCI_C_ and SCI_TL_ were longer in 8 °C LC than in 15 °C LC on both sites (all *p* < 0.05). The %ΔSkBF increased over the 50% value of the Min in all participants and both sites at 8 °C LC but not in all at 15 °C LC (Table 2).

**Table 2:** Characteristics of increased %ΔSkBF at 15 °C and 8 °C in local cooling.

		15 °C LC		8 °C LC		*p* value
	Site	SCI_C_	SCI_TL_	SCI_C_	SCI_TL_	within, between, interaction
Time_onset_ (s)	Chest	125 ± 174	81 ± 151	23 ± 28	26 ± 28	*p* = 0.06, *p* = 0.61, *p* = 0.55
	Thigh	54 ± 95	32 ± 35	30 ± 24	32 ± 17	*p* = 0.46, *p* = 0.60, *p* = 0.47
Time_peak_ (s)	Chest	270 ± 284	274 ± 208	630 ± 220^†^	730 ± 151^#^	*p* < 0.01, *p* = 0.42, *p* = 0.58
	Thigh	372 ± 251	131 ± 117	660 ± 235^†^	750 ± 196^#^	*p* < 0.01, *p* = 0.37, *p* = 0.01
Min (%baseline)	Chest	70 ± 26	50 ± 22	74 ± 44	43 ± 23^#^	*p* = 0.70, *p* = 0.07, *p* = 0.25
	Thigh	62 ± 17	52 ± 17	36 ± 18^†^	34 ± 9^#^	*p* < 0.01, *p* = 0.29, *p* = 0.34
Max (%baseline)	Chest	171 ± 112	94 ± 31	260 ± 155^†^	206 ± 111^#^	*p* < 0.01, *p* = 0.19, *p* = 0.63
	Thigh	106 ± 34	81 ± 26	182 ± 126	138 ± 63^#^	*p* = 0.01, *p* = 0.22, *p* = 0.67
Amp (%baseline)	Chest	101 ± 101	45 ± 24	186 ± 125^†^	163 ± 117^#^	*p* < 0.01, *p* = 0.35, *p* = 0.47
	Thigh	44 ± 31	29 ± 19	146 ± 117^†^	104 ± 58^#^	*p* < 0.01, *p* = 0.28, *p* = 0.51
Mean (%baseline)	Chest	125 ± 78	71 ± 30	196 ± 123^†^	140 ± 72^#^	*p* < 0.01, *p* = 0.14, *p* = 0.93
	Thigh	84 ± 23	62 ± 22	136 ± 95	98 ± 49	*p* = 0.02, *p* = 0.15, *p* = 0.64
Increase in SkBF (*N* = 9)	Chest	7/9	6/9	9/9	9/9	
	Thigh	4/9	5/9	9/9^†^	9/9^#^	

The characteristics of increased %change (Δ) in skin blood flow (SkBF) during local cooling (LC; 15 °C and 8 °C stage) in participants with cervical cord injury (SCI_C_) and thoracolumbar spinal cord injury (SCI_TL_) are shown as the mean ± SD. Time_onset_, a time to the onset of the increase in %ΔSkBF during each LC stage; Time_peak_, time to peak from the Time_onset_. Min, the lowest values before the onset of increased %ΔSkBF during each LC stage; Max, the maximum value during each LC stage; Amp, the amplitude calculated as Max–Min; Mean, the averaged value of each increased response after the onset; Increase in SkBF, number of participants in whom %ΔSkBF increased over 50% of Min. *P* values of ANOVA are shown in order of within factor (Temperature), factor of between participants (Group), and interaction of Temperature and Groups in SCI_C_ and SCI_TL_. ^†, #^, compared between 15 °C vs 8 °C LC in SCI_C_ and SCI_TL_, respectively. Significance level is *p* < 0.05.

### Body temperature and cardiovascular responses

The T_SL_ and T_S_ were comparable between the groups throughout the experiment in the chest and thigh (Table 3). The HR on the chest and thigh in participants with SCI_C_ remained unchanged throughout the experiment and SCI_TL_, respectively (Table 3). The MAP also showed no significant differences between SCI_C_ and SCI_TL_ and no changes among stages in both sites.

**Table 3:** Body temperature and cardiovascular responses.

			SCI_C_			SCI_TL_		*p* value
	Site	baseline	15 °C	8 °C	baseline	15 °C	8 °C	within, between, interaction
T_SL_ [°C]	Chest	36.3 ± 0.6	36.3 ± 0.6	36.2 ± 0.6	36.7 ± 0.4	36.4 ± 0.4	36.4 ± 0.6	*p* = 0.60, *p* = 0.18, *p* = 0.26
	Thigh	36.3 ± 0.5	36.4 ± 0.5	36.3 ± 0.5	36.6 ± 0.6	36.6 ± 0.4	36.5 ± 0.5	*p* = 0.44, *p* = 0.24, *p* = 0.41
T_S_ [°C]	Chest	32.7 ± 0.5	32.5 ± 0.6	32.6 ± 0.6	32.0 ± 1.2	32.0 ± 1.3	32.0 ± 1.3	*p* = 0.65, *p* = 0.23, *p* = 0.42
	Thigh	32.6 ± 0.5	32.3 ± 0.4	32.5 ± 0.5	32.0 ± 1.2	31.8 ± 1.3	31.8 ± 1.3	*p* = 0.09, *p* = 0.20, *p* = 0.60
HR [beats/min]	Chest	66 ± 16	66 ± 16	66 ± 15	76 ± 20	75 ± 20	77 ± 21	*p* = 0.37, *p* = 0.31, *p* = 0.75
	Thigh	68 ± 15	67 ± 15	66 ± 15	79 ± 17	77 ± 17	78 ± 19	*p* = 0.01, *p* = 0.25, *p* = 0.23
MAP [mmHg]	Chest	80 ± 13	81 ± 13	80 ± 12	91 ± 12	90 ± 12	90 ± 11	*p* = 0.66, *p* = 0.19, *p* = 0.40
	Thigh	73 ± 8	74 ± 10	74 ± 9	89 ± 12	89 ± 12	90 ± 11	*p* = 0.67, *p* = 0.01, *p* = 0.71

Sublingual temperature (T_SL_, °C), mean skin temperatures (T_S_, °C), Heart rate (HR, beats/min) and mean arterial pressure (MAP, mmHg) are shown as representative values at 33 °C (baseline), 15 °C, and 8 °C as the mean of the last 5 min for each stage. The values are presented as the mean ± standard deviation (SD) in participants with cervical spinal cord injury (SCI_C_) and those with thoracolumbar spinal cord injury (SCI_TL_). *P* values of ANOVA are shown in order of within factor (Time), factor of between participants (Group), and interaction of Time and Group in SCI_C_ and SCI_TL_.

## Discussion

The main findings of this study were as follows: (1) increased %ΔSkBF was observed at 8 °C LC in all participants and sites; thus, both intact sensory neurons and sympathetic nerve activity from the central nervous system were not necessary to initiate it, (2) the %ΔSkBF on the chest showed a significant increase from the baseline, but not on the thigh, indicating that this could be explained with the state of the sensory nerve, which was disrupted from the central nervous system in the thigh but not in the chest. This is the first study to observe increased %ΔSkBF in the non-acral skin area by LC in individuals with SCIs.

### Initiation of the increased SkBF

SkBF is determined by the balance between vasodilation and vasoconstriction activities [17, 18]. As the MAP remained unchanged throughout the present protocol, the increase or decrease in SkBF suggested cutaneous vasodilation or vasoconstriction, respectively. The observed increase in %ΔSkBF on both the chest and thigh at 8 °C LC in both groups (Figs. 2, 3, and Tables 2, 3) suggests that the trigger for this response involves local mechanisms such as dilators released from endothelial cells of cutaneous blood vessels or from sensory nerves. Since all participants had complete SCIs, it appears that the increased %ΔSkBF is not governed by central nervous drive. Yamazaki et al. [19] showed that LC stimulus of 24 °C produced a cutaneous vasodilator response in the early stage of cooling on the skin surface area where bretylium was infiltrated by an iontophoresis technique. Hodges et al. [20] reported that nitric oxide synthase (NOS) inhibitors suppressed the increase in SkBF during early cooling. These results indicate that NO is released within the vascular endothelium during LC of the skin. Therefore, vascular endothelium function could have triggered the increased %ΔSkBF in this study, although this has not been confirmed yet in SCIs.

### Potential for modifying increased SkBF through intact sensory neurons

SkBF and CVC responses on the chest in both groups (sensate) were greater than those on the thigh (non-sensate) (Figs. 2, 3) because SkBF on the chest exceeded the baseline but that on the thigh did not. Transient receptor potential channels can detect a low temperature of <17 °C [21–23], can act as a primary vascular cold sensor, and can first induce vasoconstriction due to an increased release of noradrenaline from the sympathetic nerve ending. It might then mediate the vasodilation response by involving a release of calcitonin gene-related peptide, substance P, and NOS-induced NO from the nerve endings of C-fibers [24]. Therefore, intact sensory neurons might strengthen the response of the increased SkBF, although no studies have examined whether cutaneous vasodilation activity is related to CIVD in humans. If vasodilation activity is greater than vasoconstriction activity [8, 18], CIVD is observed.

### Modification of the increased SkBF by the sympathetic nervous system

An increase in the concentration of noradrenaline has suppressed the CIVD response during cold immersion of fingers at 5 °C [25]. Hodges et al. [13] also suggested that neural activity was consistently reduced before CIVD, and sympathetic nervous system withdrawal directly contributes to CIVD onset. However, SkBF and CVC levels on the thigh did not exceed baseline values during 8 °C LC whereas they increased on the chest in both groups (Figs. 2, 3C, D). Although the sympathetic nervous system on the chest is intact in SCI_TL_, while not in SCI_C_, the responses between groups did not differ. The integrity of the sympathetic nervous system appears to play a minor role in the modification of the increased SkBF.

### Vasoconstriction during LC on the thigh

Herein, the %ΔSkBF decreased before CIVD was observed on the thigh, where the sensory nerve was not intact and activities of sympathetic nervous control were disrupted. As previously reported, after administration of bretylium, which inhibits catecholamine release from the nerve endings, by iontophoresis, LC stimulation at 24 °C or 29 °C increased SkBF and then decreased it. Hence, the vasoconstrictive effects of noradrenaline would be necessary only during early phase of LC [19, 26, 27]. Thus, the main mechanism of cutaneous vasoconstriction induced by LC on the thigh could involve the increased action of noradrenaline secreted from sympathetic nerve terminals by local mechanisms such as the mediation of Rho kinase [28, 29], non-catecholamine constriction by reduced NO, or by some actions from sensory nerve terminals [19, 20, 26, 29].

### Limitations

Criticisms of the small sample size are inevitable. However, this study found a significant difference in the patterns of SkBF between the sites. It is possible that responses in the paralyzed region could vary based on the time elapsed since injury. The variability in the time since injury remained unsolved. We did not measure any electrical activities related to indexes of skin sympathetic nervous activity in the present participants.

## Conclusion

An increase in SkBF was observed in the non-acral area (chest and thigh) by a LC stimulus (8 °C) in all participants with SCIs. The central neural mechanisms do not trigger the increase, and local mechanisms may be responsible for the changes, although the response is modified by the intact/disrupted state of the sensory nerves. The increased SkBF during LC suggests that CIVD is expressed even in SCI_C_ and SCI_TL_ and that the defense mechanism against frostbite exists in paralyzed areas.

### Supplementary information

Supplemental Figure Legends

Supplemental Figure_1

Supplemental Figure_2

Dataset

## Data Availability

The datasets used and/or analyzed during the current study are available from the corresponding author on reasonable request.
