# Supplementary material for: Observations of cold-induced vasodilation in persons with spinal cord injuries
Source: Spinal Cord. 2024 Feb 22;62(4):170–7. doi: 10.1038/s41393-024-00960-3 (PMC11003866; doi:10.1038/s41393-024-00960-3)
Supplement: Supplementary file 1 — Supplemental Figure Legends [file 41393_2024_960_MOESM1_ESM.docx]

**Supplemental Figure Legends**

Supplemental Figure 1. Skin blood flow (%Δ) during local cooling (LC) is shown as per participant in cervical and thoracolumbar spinal cord injuries (SCI_C_ and SCI_TL_) from 1 min before to the end of 15 ºC and 8 ºC stage on the chest.

Supplemental Figure 2. Skin blood flow (%Δ) during local cooling (LC) is shown as per participant in cervical and thoracolumbar spinal cord injuries (SCI_C_ and SCI_TL_) from 1 min before to the end of 15 ºC and 8 ºC stage on the thigh.
