# Supplementary figures and images for: Observations of cold-induced vasodilation in persons with spinal cord injuries
Source: Spinal Cord. 2024 Feb 22;62(4):170–7. doi: 10.1038/s41393-024-00960-3 (PMC11003866; doi:10.1038/s41393-024-00960-3)

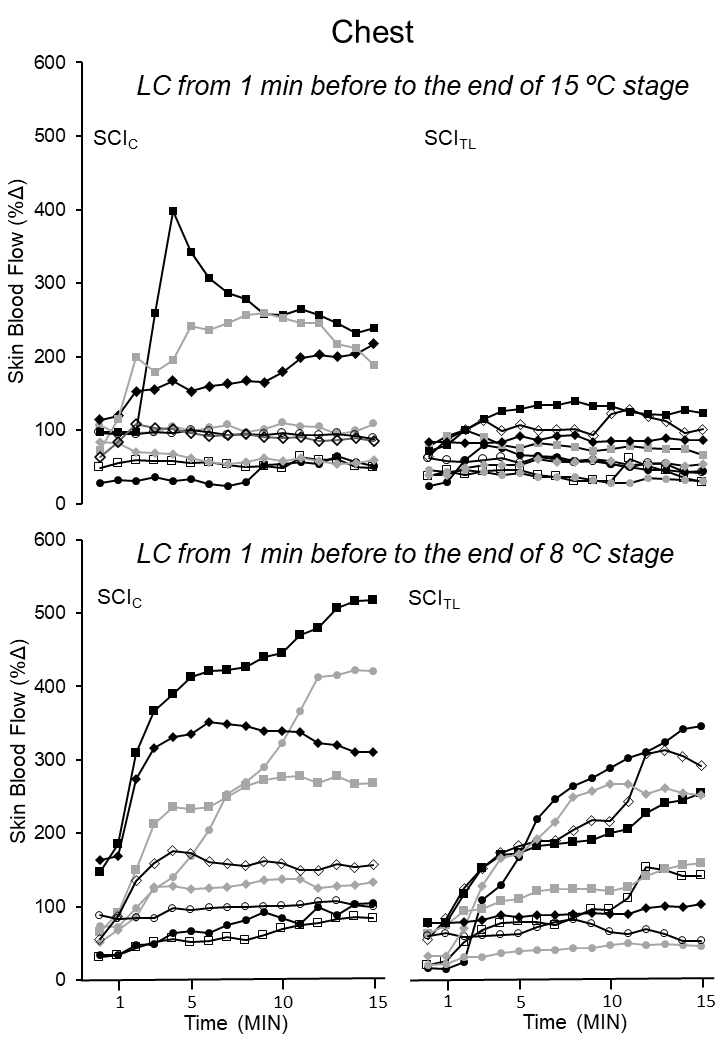

Supplement: Supplementary file 2 — Supplemental Figure_1 [file 41393_2024_960_MOESM2_ESM.tif]

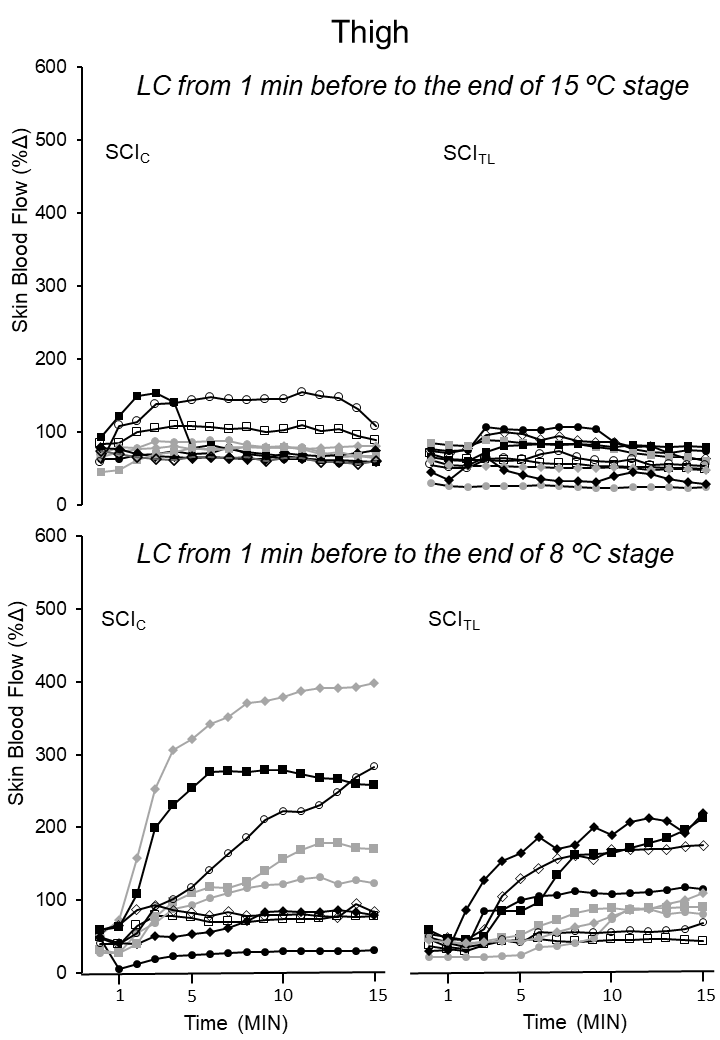

Supplement: Supplementary file 3 — Supplemental Figure_2 [file 41393_2024_960_MOESM3_ESM.tif]
